# Mental health of Portuguese nurses in 2024: what has changed since 2017? A cross-sectional observational comparative study

**DOI:** 10.1177/17449871261419412

**Published:** 2026-02-26

**Authors:** Paulo Seabra, Joaquim Oliveira Lopes, Ezequiel Pessoa, Manuel Capelas

**Affiliations:** Associate Professor on Mental Health, Nursing Research Innovation and Development Centre of Lisbon (CIDNUR), School of Nursing, University of Lisbon, Lisbon, Portugal; Associate Professor on Mental Health, Nursing Research Innovation and Development Centre of Lisbon (CIDNUR), School of Nursing, University of Lisbon, Lisbon, Portugal; Assistant Professor, Nursing Research Innovation and Development Centre of Lisbon (CIDNUR), School of Nursing, University of Lisbon, Lisbon, Portugal; Associate Professor on Palliative Care, Universidade Católica Portuguesa, Faculty of Health Sciences and Nursing, Lisbon, Interdisciplinary Health Research Centre (CIIS), Lisbon, Portugal

**Keywords:** cross-sectional study, healthcare workforce, mental health perception, nurses, occupational stress, Portugal

## Abstract

**Background:**

Nurses’ mental health has become an increasingly pressing concern worldwide because of its impact on health systems, patient health outcomes, job satisfaction, and workforce attrition.

**Aim:**

This study updates and compares findings from a national survey initially conducted in 2017 to assess the mental health of Portuguese nurses and examine associations with socio-professional variables.

**Methods:**

This cross-sectional observational study was expanded to include 1894 nurses working in hospitals, primary care, and other settings. The General Health Questionnaire-24 was used to assess mental health perception.

**Results:**

Results indicate a substantial decline in mental health perceptions across all indicators compared with 2017. Participants reported more negative assessments of overall mental health, with notable increases in somatic symptoms, anxiety and insomnia (the most affected domain), social dysfunction, and severe depression. Moreover, participants reported increased use of psychotropic drugs. Protective factors identified include specialised training, increased time off (particularly weekends), and engagement in sports or hobbies.

**Conclusions:**

Being the largest group in Portugal’s healthcare workforce, and reflected globally, nurses play a pivotal role in the health system. Their mental well-being directly impacts patient care quality and safety. These findings support the implementation of targeted strategies to safeguard nurses’ mental health and enhance healthcare delivery.

## Introduction

Nurses’ mental health is increasingly recognised as a critical concern in occupational health and public health literature (Squires et al., 2025). Even before the COVID-19 pandemic, evidence showed that nurses dealt with high levels of stress, physical and emotional exhaustion, and significant moral burden (Sagherian, 2020), with detrimental effects on their mental health and, consequently, on job satisfaction, workforce retention, and the safety and quality of patient care.

In Portugal, Seabra et al. (2019) conducted a national study in 2017 on the mental health of Portuguese nurses with the participation of 1264 nurses. The results revealed that around 66% of participants had a negative perception of their mental health; 22.2% had severe symptoms of depression, 76% had significant symptoms of anxiety, 71.6% had somatisation- for example headaches, and 94.1% had signs of social dysfunction- for example satisfaction with the completion of assigned tasks. These results were associated with factors such as older age, longer professional experience, long shifts, and working in hospital settings. On the other hand, sleep quality, family support, and leisure were identified by the authors as protective factors. Several subsequent studies have shown that the COVID-19 pandemic has accentuated a wide range of stressors, with increased damage to the mental health of healthcare professionals in general, and nurses in particular (Costa et al., 2023; Pinho et al., 2021; Sampaio et al., 2020).

Understanding the evolution of Portuguese nurses’ mental health is essential for formulating and adapting preventive and intervention strategies that respond to the real needs of the present, ensuring the quality and safety of care and the sustainability and quality of the country healthcare system. Portugal has a public national health service that provides universal coverage; however, persistent structural challenges affect nursing practice. These include low salaries, a nurse-to-population ratio below recommended levels leading to high patient-to-nurse ratios, work overload, high turnover and attrition, and a prevailing sense of insecurity in the workplace (Organisation for Economic Co-operation and Development [OECD], 2025). These factors, associated with the demanding nature of nursing practice and the need to manage complex situations such as death, dependency, trauma, loneliness, and suffering, may lead nurses to absorb difficult-to-manage emotions (Missouridou, 2017), which can contribute to psychological distress.

In this sense, after a first study carried out in 2017 (Seabra et al., 2019), the Project ‘The Mental Health of Portuguese Nurses’ was assumed as a longitudinal project with cross-sectional observational studies in regular periods. Due to the expression of the phenomenon, it is necessary to reassess it. This study updates knowledge on the mental health of Portuguese nurses in 2024 and compares this new data with the results of the 2017 study, assessing the evolution of the mental health of these healthcare professionals, as well as its relationship with socio-professional variables: age, sex, household composition, years of professional experience, professional practice context, training, work schedule, workload, health status, lifestyle. The following questions were formulated:

How has Portuguese nurses’ perception of their somatic symptoms, anxiety, social function, mood, sleep patterns, and fatigue evolved?
What are the differences in Portuguese nurses’ perception of their mental health in relation to socio-professional variables?
What risk factors and protective factors for their mental health are now identified by Portuguese nurses?

## Methodology

### Study design

This was an observational, cross-sectional and comparative study conducted within a quantitative research framework (Gray and Grove, 2021). The reporting of results adhered to the STROBE (Strengthening the Reporting of Observational Studies in Epidemiology) guidelines (Vandenbroucke et al., 2007).

### Data collection

Data were collected online between 1 February and 30 April 2024. Participants were invited to participate via newsletters distributed by the Portuguese Nurses Association (Ordem dos Enfermeiros – OE).

### Participants

The study population consisted of all nurses registered with the OE as of 31 December 2023, totalling 83,538 professionals, representing an increase of approximately 20.2% compared with the previous study, with registration serving as the sole inclusion criterion. Access to the questionnaire was provided through a hyperlink included in the study announcement. This announcement presented essential study information, with further details accessible upon clicking the link. Those who provided informed consent were directed to the questionnaire and, upon submission, were considered eligible and included in the study. The sole exclusion criterion was not being a nurse. A convenience sample was used, comprising 1894 participants, representing an increase of 49.8% compared with the previous study (*n* = 1264). This ensured a margin of error of 2.23% with a 95% confidence interval. The sample size was not predetermined, but the goal was to exceed the number of respondents as compared to the first wave.

Nursing education in Portugal is structured through a 4-year Bachelor of Nursing programme (240 European Credits Transfer System ECTS), which integrates theoretical coursework with progressive clinical placements across diverse healthcare settings. Postgraduate training is primarily delivered at the master’s level, where programmes in nursing specialties typically range from 1.5 to 2 years. These advanced degrees provide specialised preparation in areas such as medical–surgical nursing, mental health, community health, rehabilitation, and child health, aligning clinical competencies with national regulatory standards. In all areas, nurses are exposed to emotional distress but only recently nursing education in Portugal has gradually begun to integrate components relevant to spiritual care, grief, and holistic care – though not always as explicit, standalone units. This could be a protective factor for mental health in the future (Afonso et al., 2023).

### Data collection instrument

The questionnaire used in this study was an updated version of the instrument previously applied in 2017 (Seabra et al., 2019). It included quantitative variables related to socio-demographic and professional characteristics, and lifestyle habits, and introduced a new variable: *receiving psychological support*. As in the previous study, the questionnaire was developed by the authors to include variables considered important for Portuguese nursing labour conditions. To assess participants’ mental health perception, the General Health Questionnaire-24 (GHQ-24) was employed (Seabra et al., 2021). This self-report instrument is adapted from the Portuguese version of the GHQ-28 (Pais-Ribeiro et al., 2015) and evaluates the current state of mental health (i.e. symptom perception), rather than stable traits or formal diagnoses. Each item is rated on a four-point ordinal scale (0–3), resulting in a total score ranging from 0 to 72. A cut-off score of 20.5 indicates the threshold for clinical psychiatric referral (Seabra et al., 2021), with higher scores reflecting poorer mental health.

The GHQ-24 comprises four subscales, which measure symptom dimensions rather than distinct disorders. The subscales and their respective ranges and cut-off scores are as follows: Somatic Symptoms (Items 1–6): range 0–18, cut-off: 4.5; Anxiety and Insomnia (Items 7–13): range 0–21, cut-off: 5.5; Social Dysfunction (Items 14–18): range 0–15, cut-off: 4.5; Severe Depression (Items 19–24): range 0–18, cut-off: 4.5.

### Data analysis

Data were analysed using Microsoft Excel ((Microsoft Corporation, Redmond, WA, United States) and IBM SPSS Statistics, version 29 for Windows (IBM Corp., Armonk, NY, United States). Both descriptive and inferential statistics were applied. Based on the assumption of normality, appropriate descriptive measures (mean, median, standard deviation, interquartile range) and statistical tests were selected. The following tests were used: the Mann–Whitney *U*-test for comparisons between two independent groups (ordinal/continuous variables); the Kruskal–Wallis test for comparisons among three or more independent groups (ordinal/continuous variables); Spearman’s correlation (r) for assessing relationships between continuous or ordinal variables; chi-square test (or Fisher’s exact test, when applicable) for associations between categorical variables. A significance level of *p* < 0.05 was adopted. Analyses were first performed on the 2024 dataset, and comparisons with 2017 data were conducted where appropriate.

To identify predictors of mental health outcomes, two binary logistic regression models using the forward (Wald) method were applied: one for the total sample and another specifically for nurses working shifts. Subsequent analyses were conducted on the GHQ-24 subscales. The use of a previously validated instrument minimised the risk of measurement bias. No missing data were anticipated due to the design of the online questionnaire.

### Ethics approval and consent to participate

Following all aspects of the Declaration of Helsinki this study received approval from the Ethics Committee of the Nursing School of Lisbon (Process No.: 4718/2023). Participants accessed the online questionnaire after reading detailed study information and providing informed consent. Consent was mandatory to proceed with participation. Individuals who opted not to participate could exit without submitting their responses. Given the potential psychological sensitivity of certain items, information on mental health support resources was provided to all participants.

## Results

### Participants

A total of 1894 nurses participated in the study – an increase of 630 participants compared to the 2017 survey, which included 1264 nurses.

When characterising the sample (Table 1), some variables showed consistency between the two cohorts, such as age. However, the 2024 sample included a slightly younger group overall: the first quartile in 2024 was up to 29 years of age, compared to 31 years in 2017. Consistency was also observed in the mean years of professional experience; however, the mode shifted to 1 year, reflecting the higher proportion of younger nurses in the current cohort. This is further supported by the increase in participants with up to 10 years of professional experience, rising from 36.4% in 2017 to 40.6% in 2024. The variable reflecting employment in more than one workplace also remained stable across the two samples.

**Table 1.** Demographic, professional and social characteristics 2024 versus 2017.

Variable	2024 % (*n*) Mean (SD) Median (IQR)	2017 % (*n*) Mean (SD) Median (IQR)	Analysis
Age, years	37.95 (10.5)/38 (16)	38.22 (9.57)/36 (14)	*U* = 1,194,178.000; *p* = 0.990
Sex			
Feminine	87.8% (1663)	83.2% (1052)	*X*^2^(1) = 13.739; *p* < 0.001
Masculine	12.1% (229)	16.8% (212)	
Household size	2.10 (1.24)/2 (2)	2.88 (1.13)/3 (2)	*U* = 761,456.500; *p* < 0.001
Living alone	8.4% (159)	12.3% (156)	*X*^2^(1) = 13.151; *p* < 0.001
Years of professional experience	15.19 (10.53)/14 (17) Mode = 1	15.20 (9.51)/13 (14) Mode = 11	*U* = 1174,269.500; *p* = 0.365
Professional practice context			
Hospital	67.7% (1283)	60% (759)	*X*^2^(2) = 19.635; *p* < 0.001
Primary healthcare	17.8% (338)	22.2% (280)	*X*^2^(1) = 0.503; *p* = 0.478
Other contexts	14.4% (273)	17.8% (225)	*X*^2^(1) = 9.403; *p* = 0.002
Works in more than one location	26.6% (510)	25.8% (326)	*X*^2^(1) = 0.503; *p* = 0.478
Specialist nurse title	39.8% (753)	45.3% (572)	*X*^2^(1) = 9.403; *p* = 0.002
Area of specialisation (*n* = 753)			
Medical-surgical	38.3% (288)	16.4% (94)	
Mental health and psychiatric	14.3% (108)	32.5% (186)	
Child and paediatric health	9.7% (73)	14.2% (81)	
Rehabilitation	10.5% (79)	12.6% (72)	
Community health	7.6% (57)	13.6% (78)	
Maternal and obstetric health	19.7% (148)	9.8% (56)	

IQR: interquartile range; SD: standard deviation; U: Mann–Whitney test.

Several differences were identified in the 2024 sample compared with 2017, including a higher proportion of female participants, a decrease in the number of individuals living in the same household or alone, an increase in the proportion working in hospital settings and a decrease in the proportion of nurses with a specialisation.

### Working conditions

Regarding working conditions and environment (Table 2), consistency was noted across both samples in several variables, including: years working shifts, weekly working hours (combining main job and additional work), number of night shifts per month, and perceived workload during night shifts. However, statistically significant differences emerged in the 2024 sample compared with 2017. Higher values were observed for the proportion of participants reporting shift work, number of days off per month, perceived workload during morning shifts, and inability to rest during night shifts. Lower values were noted for the number of night shifts per month and the number of free weekends per month.

**Table 2.** Characterisation of working conditions in 2024 versus 2017.

Variable	2024 % (*n*) Mean (SD) Median (IQR)	2017 % (*n*) Mean (SD) Median (IQR)	Analysis
Work in shifts	67.7% (1283)	58.5% (740)	*X*^2^(1) = 27.844; *p* < 0.001
Years working shifts (*n* = 1283)	11.15 (9.09)/8 (13)	11.23 (8.32)/10 (12)	*U* = 452,726.000; *p* = 0.275
Number of weekly working hours	43.97 (14)/40 (13)	43.90 (9.87)/40 (15)	*U* = 1,216,470; *p* = 0.236
Number of monthly working nights (*n* = 1112)	6.46 (2.80)/6 (3)	6.73 (2.26)/7 (3)	*U* = 327,108.000; *p* < 0.001
Number of monthly days off	6.70 (2.71)/7 (3)	6.03 (2.71)/6 (4)	*U* = 103,7921; *p* ⩽ 0.001
Number of monthly free weekends	1.69 (1.45)/1 (2)	1.91 (1.58)/1 (3)	*U* = 1100423.500; *p* ⩽ 0.004
Workload during shifts			
Morning (*n* = 1813) – very heavy/extremely heavy	73.4% (1331)	67% (491)	*U* = 625,293.500; *p* = 0.012
Night (*n* = 1233) – very heavy/extremely heavy	54.7% (675)	50.8% (342)	(*U* = 395,597.500; *p* = 0.075)
Opportunity to rest during night shifts (*n* = 1151) *Occasionally/never*	73.2% (842)	70.2% (483)	*X*^2^(3) = 9.444; *p* = 0.024

### Further analyses

Regarding the health status, lifestyles, and sleep patterns of nurses (Table 3), some differences were found compared to 2017. Negative perceptions increased for physical health, emotional/psychological health, the need for more sleep between morning and afternoon shifts and tiredness at the end of the day. Conversely, reductions were observed in the practice of physical activity or hobbies, perception of healthy eating, morning vitality, and ease of falling asleep. No differences were found regarding hours of sleep or need for more sleep between night shifts.

**Table 3.** Characterisation of nurses’ health status, lifestyles and sleep patterns.

Variable	2024 % (*n*) Mean (SD) Median (IQR)	2017 % (*n*) Mean (SD) Median (IQR)	Analysis
Health status			
Self-reporting of illness	45% (853)	38% (480)	*X*^2^(1) = 87.632; *p* < 0.001
Perception of being physically healthy			
Occasionally/never	61.2% (1158)	25.6% (324)	*U* = 763,410; *p* < 0.001
Always/Almost always	38.8% (736)	74.4% (940)	
Perception of being emotional/psychological healthy			
Occasionally/Never	44.6% (846)	28% (354)	
Always/Almost always	55.4 (1048)	71% (910)	*U* = 1002,772; *p* < 0.001
Lifestyles			
Practise of physical activity/hobby	44% (834)	56% (628)	*X*^2^(1) = 9.733; *p* = 0.002
Perception of healthy eating			
Occasionally/Never	50.9% (964)	28.9% (365)	*U* = 946,856.500; *p* < 0.001
Always/Almost always	49.1% (930)	71.1% (899)	
Sleep patterns			
Number of daily hours of sleep	6.39 (0.98)/ 6 (1)	6.39 (0.99) / 6 (1)	*U* = 1152,868; *p* = 0.201
Perception of being able to fall asleep easily anywhere			
Probably yes/Definitely yes	28.6% (541)	32.2% (407)	*U* = 1,144,707; *p* = 0.032
Maybe	12.7% (240)	13.2% (167)	
Probably no/Definitely no	57.7 (1113)	54.6% (690)	
Perception of more vitality in the morning and tiredness at the end of the day	40.1% (760)	45.7% (577)	*U* = 1,116,978.500; *p* = 0.001
Perception of sleep quantity between shifts			
Morning – Need to sleep more or much more	60.6% (1099)	49.1 (363)	*U* = 572,075; *p* < 0.001
Afternoon – Need to sleep more or much more	40.8% (595)	31.3% (227)	*U* = 460,690.500; *p* < 0.001
Night – Need to sleep more or much more	74.4% (915)	72.4% (489)	*U* = 400,643; *p* = 0.170

Compared to 2017, regarding the use of medication, addictive substances, and Psychological/Psychiatric follow-up (Table 4) differences were found in the following variables: consumption of anxiolytics (increase), consumption of antidepressants (increase), and consumption of sleep inducers (significant increase). There were no differences in the percentage of smokers and regular alcohol users.

**Table 4.** Characterisation of medication, addictive substances and psychological/psychiatric follow-up.

Variable	2024 % (*n*) Mean (SD) Median (IQR)	2017 % (*n*) Mean (SD) Median (IQR)	Analysis
To smoke	16.9% (321)	14.9% (188)	*X*^2^(1) = 2.414; *p* = 0.120
Alcohol consumption – Often/Always	1.3% (*n* = 24)	1.6% (20)	*U* = 1,178,435.500; *p* = 0.405
Anxiolytics	17.3% (327)	12.6% (159)	*X*^2^(1) = 12.784; *p* < 0.001
Antidepressants	17.7% (336)	11.9%(150)	*X*^2^(1) = 20.082; *p* < 0.001
Sleep inducers/hypnotics	21.6% (409)	12.3%(156)	*X*^2^(1) = 44.180; *p* < 0.001
Psychological/psychiatric follow-up in the last 30 days	21.3% (403)	Wasn’t questioned	

### Main results on mental health

There were significant variations between 2017 and 2024 in all mental health indicators (Table 5) revealing a significant worsening.

**Table 5.** Characterisation of Portuguese nurses mental health.

Variable	2024 % (*n*) Mean (SD) Median (IQR)	2017 % (*n*) Mean (SD) Median (IQR)	Analysis
Perception of Mental Health (GHQ24)			
Global (score) With a negative perception	28.31(11.79)/28(16) 74.3% (1407)	24 (11. 92)/24 (16) 59% (746)	*U* = 1,433,643.500; *p* < 0.001 +15.3%
Somatic symptomatology (score) With somatic symptomatology present	7.71 (3.6)/8 (5) 79.4% (1503)	6.85 (3.7)/6 (5) 69.5% (878)	*U* = 1,373,595.500; *p* < 0.001 +9.9%
Anxiety and insomnia (score) With anxiety and insomnia present	11.08 (4.72)/11 (6) 87.4% (1656)	9.12 (4.83)/9 (7) 76% (962)	*U* = 1,471,786; *p* < 0.001 +11.4%
Social dysfunction (score) With social dysfunction present	6.69 (2.35)/6 (3) 91.4% (1731)	6.31 (2.28)/6 (2) 88.8%% (1123)	*U* = 1,316,986.500; *p* < 0.001 +2.6%
Severe depression (score) Symptoms of severe depression present	3.29 (3.95)/2 (5) 28.8% (546)	2.70 (3.83)/1 (4) 22.4% (283)	*U* = 1,328,348.500; *p* < 0.001 +6.4%

### Socio-demographic characteristics

Regarding gender, both studies found that women have a worse perception of mental health, with a decline observed in both sexes in 2024. Specifically, in 2017, males had a median (me) score of 20 (IQR = 14.75) and females a median of 24 (IQR = 16) (*U* = 91.984.5; *p* < 0.001). In 2024, these values increased to a median of 24 (IQR = 17) for males and 28 (IQR = 16) for females (*U* = 160.151; *p* < 0.001).

Concerning age, in 2017, older age was associated with a higher perception of severe depression (r = 0.104; *p* < 0.001). However, in 2024, older age showed a weak but significant correlation with lower anxiety and insomnia (r = −0.112; *p* < 0.001) as well as less social dysfunction (r = −0.125; *p* < 0.001). No correlation was found between age and overall mental health perception (total scale) in either study. Participants aged 18 to 29 had the worst perception of mental health (me = 29; IQR = 16), and the differences between age groups were statistically significant (KW(4) = 19.948; *p* < 0.001).

### Lifestyle habits

The practice of physical activity or having a hobby is associated with better mental health (median = 25; IQR = 9) compared to those who do not engage in these activities (median = 29; IQR = 16). The 2024 data (*U* = 356.222.0; *p* < 0.001) confirm the findings from 2017. Regarding sleep duration, the relationship is even stronger. In 2024, more hours of sleep correlated with better overall mental health perception and improved scores across all subscales (r = −0.258; *p* < 0.001), consistent with the 2017 data (r = −0.245; *p* < 0.001).

Concerning smoking habits no differences were found in 2017. However, in 2024, smokers had a more negative perception of mental health (median = 29; IQR = 15.5) compared to non-smokers (median = 27; IQR = 16) (*U* = 231.847.5; *p* = 0.021). Regarding alcohol consumption, neither study found significant differences related to the frequency of drinking among nurses (*p* > 0.005).

For psychotropic drug use, in 2024, participants who reported using anxiolytics had a more negative perception of mental health (median = 36; IQR = 16) than those who did not (median = 26; IQR = 15) (*U* = 44.088.5; *p* < 0.001). Similarly, antidepressant users reported worse mental health perceptions (median = 35; IQR = 20) compared to non-users (median = 27; IQR = 16) (*U* = 170.547.5; *p* < 0.001). The same pattern was observed for users of sleep inducers/hypnotics (median = 34; IQR = 17) versus non-users (median = 26; IQR = 15) (*U* = 184.298; *p* < 0.001). This difference was also evident in the 2017 data.

### Socio-working conditions

Regarding years of service, the 2017 data showed a positive correlation between more years of service and higher levels of depression (r = 0.106; *p* = 0.004). However, in 2024, longer years of service were associated with lower levels of anxiety and insomnia (r = −0.100; *p* < 0.001) as well as less social dysfunction (r = −0.090; *p* < 0.001).

Concerning specialised training recognised by the Ordem dos Enfermeiros, there was no statistically significant difference among participants in 2017. In contrast, 2024 data revealed a significant difference: those with specialty training reported better mental health (median = 25; IQR = 17) compared to those without (median = 29; IQR = 15) [*U* = 362.897.5; *p* < 0.001]. In other words, having a nursing specialty is associated with better mental health.

Regarding work areas, nurses in the medical-surgical field had the most negative perception of mental health (median = 28; IQR = 17), whereas those working in maternal and obstetric health reported a better perception (median = 23; IQR = 15.75) [KW(5) = 12.814; *p* = 0.025]. In 2024, regarding the work setting of participants, mental health scores varied significantly across contexts: hospital (median = 28; IQR = 16), primary health care (PHC; median = 27.5; IQR = 17) and other contexts (median = 30; IQR = 16.5) [KW(2) = 10.114; *p* = 0.006]. Those working in ‘other contexts’ reported worse mental health, although no significant difference was found when comparing only hospital care and PHC (*U* = 214.138; *p* = 0.725). In 2017, there were no overall mental health differences between contexts. However, in 2017, working in ‘other contexts’ was associated with more somatic symptoms (KW(2) = 11.387; *p* = 0.003). In 2024, this group showed significantly worse scores across subscales, including anxiety and insomnia, social dysfunction and severe depression (all *p* < 0.05). When comparing hospital care and PHC in 2024, a small but significant difference was found: those working in hospital care had a worse perception of social dysfunction (*U* = 199.014.5; *p* = 0.018).

No differences were found in mental health perception between those working in a single workplace and those working in multiple workplaces in 2024 (*U* = 350.352.5; *p* = 0.808), which contrasts with 2017 data were working in multiple places was associated with better mental health perception (*U* = 134.992.5; *p* = 0.002).

Concerning shift work, no differences were observed in 2017. However, in 2024, shift workers reported a more negative perception of their mental health (median = 29; IQR = 16) compared to non-shift workers (median = 26; IQR = 16) (*U* = 353.471; *p* < 0.001), with increased anxiety, insomnia and social dysfunction (*p* < 0.001). The correlation between years of shift work and mental health perception was minimal. Notably, those with 0–5 years of shift work experienced higher anxiety, insomnia, and social dysfunction.

Regarding weekly working hours, no relationship with mental health was found in 2017. In 2024, however, a weak but significant correlation was observed: more hours worked per week were associated with worse mental health perception (r = 0.131; *p* < 0.001) across all subscales. Additionally, participants perceiving their workload as very high during any shift (morning: median = 33; IQR = 16; afternoon: median = 33; IQR = 16; night: median = 34; IQR = 14.75) reported significantly poorer mental health [KW(4) = 94.349; *p* < 0.001].

With regard to night work, in 2024, data show that the more night shifts worked, the more negative the perception of overall mental health (r = 0.081; *p* = 0.006) and across all subscales. Similarly, having the opportunity to rest during night shifts is associated with better mental health perception (KW(3) = 29.326; *p* < 0.001). Those who reported *never* having the chance to rest had worse mental health (median = 32; IQR = 17), whereas those who *always* had the opportunity to rest reported better mental health (median = 22; IQR = 14.25).

Regarding rest periods, in 2017, there was no significant relationship between the number of days off and mental health perception. However, in 2024, more days off correlated with better mental health (r = −0.092; *p* < 0.001) and more free weekends were also linked to improved mental health (r = −0.097; *p* < 0.001).

Concerning health perceptions and care, in 2024, participants who reported psychological or psychiatric follow-up in the past 30 days perceived their mental health as more impaired (median = 34; IQR = 17) compared to those without such follow-up (median = 26; IQR = 16) (*U* = 195.723.5; *p* < 0.001).

### Multivariate analysis

Using binary logistic regression with the ‘Wald forward’ method, the predictors of overall mental health perception and its specific dimensions were examined. Five statistically significant models were identified. In all models, the reference category of the dependent variable was defined as ‘positive perception’, according to the established cut-off points.

For the perception of overall mental health, the analysis identified that: (1) female nurses were 80.4% more likely to report a negative perception (Exp(B) = 1.804; 95% CI: 1.333–2.442) compared with male nurses; (2) engaging in sports activity or a hobby reduced the likelihood of a negative perception by 48.08% (Exp(*B*) = 0.519; 95% CI: 0.419–0.643); (3) being a specialist nurse reduced the likelihood of a negative perception by 29.79% (Exp(*B*) = 0.703; 95% CI: 0.555–0.890); (4) each additional hour of work per week increased the likelihood of a negative perception by 1.61% (Exp(*B*) = 1.016; 95% CI: 1.003–1.029) and (5) among shift workers, each additional hour of sleep decreased the likelihood of a negative perception by 37% (Exp(*B*) = 0.630; 95% CI: 0.539–0.738).

Regarding the perception of psychosomatic symptoms, it was identified that: (1) each ‘slack’ period decreased the likelihood of a negative perception by 4.35% (Exp(B) = 0.957; 95% CI: 0.915–1.000); and (2) each free weekend per month reduced the likelihood of a negative perception by 8.23% (Exp(B) = 0.918; 95% CI: 0.846–0.996). In relation to social dysfunction, each additional free weekend per month reduced the likelihood of a negative perception by 19.4% (Exp(B) = 0.623; 95% CI: 0.442–0.877).

For anxiety and insomnia, each year of professional practice reduced the likelihood of a negative perception by 2% (Exp(*B*) = 0.980; 95% CI: 0.967–0.993). Concerning depression, nurses working in ‘other contexts’ were 65.94% more likely to report severe depressive symptoms compared with those working in hospital settings (Exp(*B*) = 1.659; 95% CI: 1.253–2.197).

## Discussion

Given that this study adopts a national survey perspective, it is important to frame the discussion of these results in relation to the 2017 findings (Seabra et al., 2019), which at the time represented the most comprehensive study conducted with Portuguese nurses in terms of sample size and data detail, as well as in the context of relevant literature. This paper updates our understanding of Portuguese nurses’ mental health and provides insight into the evolution of factors related to their professional, living, working, and health conditions, following a traumatic period for healthcare professionals, the COVID-19 pandemic, during which nursing staff worldwide experienced alarming levels of psychological distress (Ramos-Toescher et al., 2020).

Analysing the evolution of mental health perception across various variables reveals notable changes regarding age and, correspondingly, years of professional experience. Younger nurses and those with fewer years of practice report higher levels of anxiety, insomnia and social dysfunction compared to older colleagues, this being a change from the 2017 data. This negative perception aligns with findings from other recent studies (Moura et al., 2022; Ring and Hult, 2025; Yuan et al., 2023), which highlight work demands, contractual instability and uncertain prospects – exacerbated by the COVID-19 context – as significant stressors contributing to burnout and mental overload (Moya-Salazar et al., 2023; Yuan et al., 2023). For this reason, data collection for the current study was conducted in 2024 to allow some temporal distance from the pandemic’s peak, however, these findings may be influenced by the long-term effects of COVID-19. Recent studies indicate that the psychological impact of COVID-19 on healthcare workers can persist well beyond the acute phase of infection. Healthcare workers with long COVID-19 syndrome exhibit significantly higher levels of anxiety and depression compared with those without the syndrome (Zhang et al., 2025). Similarly, a European study reported that healthcare professionals exposed to COVID-19 patients continued to experience substantial anxiety and depression, with the highest levels observed among those who had themselves contracted COVID-19 (Gaddour et al., 2025).

Older age and greater professional experience appear to serve as protective factors for mental health, enabling better coping with challenges that disproportionately affect nurses early in their careers. Regarding sex, this study found a stronger association between female participants and negative mental health perception than reported in previous burnout studies (Silva et al., 2016) and the 2017 survey (Seabra et al., 2019). These results align with broader research showing that women and young adults in the general population exhibit more pronounced psychological distress (Almeida et al., 2021; Zhang et al., 2023), and that female nursing professionals are at greater risk of mental disorders (Moura et al., 2022).

Regarding personal factors associated with better mental health, the data reinforce the 2017 findings. Regular physical activity or having a hobby are strongly associated with, and predictive of, improved mental health. This aligns with a study of Portuguese nurses showing that coping and self-distraction strategies reduce psychosocial risks and are linked to better mental health (Gomes et al., 2013), as well as findings in other healthcare professionals (Almeida et al., 2022). Another important predictive factor for better mental health is postgraduate training. Holding postgraduate qualifications at the specialisation level, which contributes to personal fulfilment, acts as a protective factor, consistent with the 2017 results (Seabra et al., 2019) and other recent studies (Yuan et al., 2023).

In terms of professional practice context, it is evident that nurses working in hospital settings report greater social dysfunction compared to those in primary care centres. This is largely due to the impact of shift and night work (Yuan et al., 2023) and the demands of increasingly high-tech environments (Yan et al., 2025), factors associated with a breakdown in psychological attachment to work (Ring and Hult, 2025). Additionally, nurses working in ‘other contexts’ show more negative mental health perceptions, potentially reflecting exposure to less structured organisations with fewer technical and human resources and less effective leadership. However, there is a lack of in-depth research exploring these settings.

Regarding shift work the findings support existing evidence that shift work is linked to poorer mental health – particularly increased anxiety, insomnia and social dysfunction, compared to non-shift workers (Lim and Kim, 2025; Paguio et al., 2020). Notably, among shift workers, those with fewer years of shift work experience report worse mental health than those with longer experience. Other studies suggest that burnout, fatigue, and sleep disturbances can affect nurses regardless of age but may impact early-career professionals more severely (Fond et al., 2023a). These results correspond with findings related to years of practice, where longer shift work experience is associated with better mental health, possibly due to greater resilience and reduced anxiety and depression over time (Vala et al., 2025).

Regarding working conditions, this study reinforces findings from previous research: longer weekly working hours are associated with poorer mental health (Almeida et al., 2021), perceptions of high workload during shifts correlate with worse mental health (Silva et al., 2016), and more days off and rest are linked to better mental health outcomes (Min and Hong. 2022; Seabra et al., 2019). Notably, there is a scarcity of studies distinguishing the effects of the number of days off versus free weekends. Consistent with Seabra et al. (2019), this study confirms that the number of free weekends has a stronger association with better mental health than the total number of days off. The positive relationship between longer sleep duration and better mental health (Min and Hong, 2022; Pereira et al., 2021) is a consistent finding across studies but is often disrupted by shift work. In terms of lifestyle habits, our data link smoking and alcohol consumption to poorer mental health as supported by other studies (Albakri et al., 2024; Fond et al., 2023a), which also report higher tobacco and coffee consumption among those working shifts longer than 7 hours. Smoking and coffee drinking may function as maladaptive coping mechanisms for psychological overload (Fond et al., 2023a).

Regarding the primary outcome, 74.3% of participants reported impaired mental health perception, representing a 15.3% increase compared to 2017. A breakdown by dimensions revealed severe depression in 28.8% of participants (+6.4%), somatic symptomatology in 79.4% (+9.9%), anxiety and insomnia in 87.4% (+11.4%) and social dysfunction in 91.4% (+2.6%). These figures substantially exceed those reported in the 2017 survey conducted by the same research group (Seabra et al., 2019) and surpass the 22% prevalence of mental disorders identified in the general Portuguese population in 2019, which itself is higher than the European Union average of 16.7% (OECD, 2023). Anxiety disorders were the most common (9%), followed by depressive disorders (6%), and alcohol/drug use disorders (4%) (OECD, 2023). The higher prevalence of depression among women in the Portuguese population aligns with the predominantly female composition of the present sample. These findings are more concerning than those reported in a study of emergency nurses in Brazil, which found a 20.5% prevalence of common mental disorders (Moura et al., 2022), and higher than the 30% of nurses identified as experiencing moderate or severe burnout (Silva et al., 2016). Using the GHQ-28 instrument, Meneses et al. (2024) reported that 69.2% of Portuguese health professionals (59.1% nurses) had negative mental health perception, close to the 74.3% observed in the current study. A study involving Portuguese physical therapists also using the GHQ-28 found lower percentages of negative mental health perceptions, particularly among those engaging in regular physical activity, supporting the present findings (Almeida et al., 2022).

Regarding psychotropic drug use, 17.3% of participants reported using anxiolytics, 17.7% used antidepressants, and 21.6% used sleep inducers – an increase compared with 2017, but still lower than the rates reported in a French sample of 899 nurses diagnosed with depression, in which usage reached 21.6%, 23.8% and 37.7%, respectively (Fond et al., 2023b), with the largest difference observed for sleep inducers. These concerning figures, reported in one of the countries with the highest rates of psychotropic drug use, do not indicate a decline in benzodiazepine consumption. Instead, they highlight a rising trend in the use of hypnotics, a pattern also observed in international studies (Ma et al., 2023). Additionally, 21.3% of participants reported receiving psychological or psychiatric counselling, similar to the 24.1% observed among French health professionals diagnosed with major depression (Fond et al., 2023b). These findings may indicate insufficient monitoring of individuals experiencing psychological distress, given the much lower proportion of those who reported impaired mental health perception. Notably, participants under professional monitoring reported poorer mental health perceptions, potentially reflecting increased self-awareness and engagement in self-care.

There was a concerning increase (+7%) in participants reporting at least one diagnosed disease (45%) and a decrease (−16.7%) in those perceiving themselves as psychologically or emotionally healthy. When compared with the 74.3% of participants presenting negative GHQ-24 scores, this discrepancy may suggest a tendency to underestimate or undervalue one’s own mental health status. This may be related to difficulties in managing emotions inherent to the nursing profession, particularly due to regular exposure to patients’ and families’ trauma. Nurses may not fully recognise the impact of cumulative trauma, may experience difficulties in articulating and processing emotionally demanding experiences, and may be less likely to discuss these experiences openly (Missouridou, 2017), which may contribute to sustained psychological distress.

Finally, the indicators of adverse mental health outcomes should be interpreted within the specific context of nursing practice in Portugal. A persistent shortage of nurses, nurse-to-patient ratios below recommended levels, and work overload (OECD, 2025), together with limited autonomy, outdated organisational structures, and the underutilisation of advanced skills, characterise daily nursing practice. Within this context, factors such as unbalanced working hours, insufficient rest breaks, limited empathetic leadership, unsupportive work environments, and restricted career development opportunities have been identified as contributing to lower job satisfaction, higher burnout, and poorer nursing practice environments (Lucas et al., 2025).

### Implications for practice

These findings highlight the need to implement structured mental health support strategies within nursing practice, given the emotionally demanding nature of the profession, sustained exposure to patients’ and families’ trauma, and high levels of occupational strain. Negative mental health indicators may remain under-recognised due to sub-diagnosis, limited continuity of mental healthcare, and professional norms that encourage emotional restraint. As a result, psychological distress among nurses may persist without timely identification or intervention. To address these challenges, clinical settings should integrate routine mental health screening, trauma-informed supervision, and regular emotional debriefing sessions into nursing practice. Additionally, ensuring clear referral pathways and follow-up in mental health services, alongside organisational policies that normalise emotional expression and help-seeking, may facilitate earlier intervention, reduce cumulative distress, and support nurses’ psychological well-being while safeguarding quality of care.

### Strengths and limitations

Given the sample size and the calculation of the margin of error relative to the population, some confidence can be placed in the generalisability of these findings. This study is comprehensive, as it explores differences across a wide range of personal and work-related variables. Another strength is that the scale is validated, which enables reliable comparisons with other populations. Nevertheless, the use of a convenience sample should be considered a limitation, as well as the potential for self-selection bias, with participants possibly experiencing particularly challenging periods in terms of their mental health being more likely to respond. Another limitation of the study is that, while the instrument employed is comprehensive, it does not capture all relevant dimensions, and structural or organisational factors such as staffing ratios and workload were not specifically examined, which may overemphasise individual responsibility for mental health outcomes that are largely shaped by systemic conditions.

## Conclusion

This study, the largest ever conducted on the mental health of Portuguese nurses, reveals a clear deterioration in mental health perceptions across all indicators compared to the previous survey conducted 7 years earlier. There is a generally more negative view of mental health, with notable worsening in somatic symptoms, anxiety and insomnia (which showed the greatest increase), social dysfunction and severe depression. These outcomes can be linked to related factors: nurses report poorer sleep quality, signs of fatigue (such as feeling the need for more sleep after any type of shift), and a high perceived workload.

The sample included a younger cohort of nurses, among whom mental health perceptions were particularly negative. Conversely, longer professional experience was associated with lower anxiety levels and reduced social dysfunction, indicating that age and years of practice act as protective factors. Consistent with other research, working in hospital settings, rotating shifts and an increased number of night shifts were linked to poorer mental health. However, several protective factors also emerged, including specialised training, more days off (especially weekends), regular physical activity, and engaging in hobbies. Other studies have also highlighted that male healthcare professionals tend to perceive their mental health as better than their female counterparts. In some societies, this difference has been linked to the roles women play, which often involve high social expectations to fulfil family responsibilities alongside demanding professional obligations.

This study contributes to a deeper understanding of the challenges faced by Portuguese nurses and provides valuable evidence to inform strategies and policies aimed at supporting their mental health.

**Key points for policy, practice and/or research**

– Portuguese nurses are sleeping worse, feeling more work overload, and their mental health has worsened compared to 2017.
– Almost three-quarters (74.3%) of nurses perceive their mental health negatively. About 91.4% of nurses perceive social dysfunction, 87.4% experience anxiety and insomnia, 79.4% experience somatic symptoms, and 28.8% experience severe depression.
– Women of all ages and younger professionals experience the most negative mental health perceptions.
– Professional experience, postgraduate training, and regular physical activity or hobbies contribute to better mental health.
– Further research should examine structural factors such as workload, staffing, shift patterns, and organisational support to inform effective organisational and policy-level changes.
– Understanding the challenges faced by Portuguese nurses is essential to guide strategies and policies supporting their mental health.

## References

[bibr1-17449871261419412] Afonso A Sitefane S Rabiais I , et al. (2023) Spiritual care in the undergraduate nursing degree in Portugal. Education Sciences 13: 273. DOI: 10.3390/educsci13030273.

[bibr2-17449871261419412] Albakri U Smeets N Kant I , et al. (2024) Strategies that nurses working irregular night shifts use to improve sleep quality: A qualitative study among good and poor sleepers. Journal of Advanced Nursing 80: 2038–2050. DOI: 10.1111/jan.15962. 37964484

[bibr3-17449871261419412] Almeida MH Marques C Simões A (2021) Burnout em profissionais de saúde: Uma análise centrada nas mulheres em contexto hospitalar português. Revista da Escola de Enfermagem da USP 55: e03740.

[bibr4-17449871261419412] Almeida LC Grilo A Carolino E , et al. (2022) Relationship between physical activity levels of Portuguese physical therapists and mental health during a COVID-19 pandemic: Being active is the key. Frontiers in Public Health 10: 986158. DOI: 10.3389/fpubh.2022.986158. 36388292 PMC9665837

[bibr5-17449871261419412] Costa A Almeida TC Fialho M , et al. (2023) Mental health of healthcare professionals: Two years of the COVID19 pandemic in Portugal. International Journal of Environmental Research and Public Health 20: 3131. DOI: 10.3390/ijerph20043131. 36833822 PMC9968046

[bibr6-17449871261419412] Fond G Lucas G Boyer L (2023a) Health-promoting work schedules among nurses and nurse assistants in France: Results from nationwide AMADEUS survey. BMC Nursing 22: 1–8. DOI: 10.1186/s12912-023-01403-9. 37537611 PMC10399037

[bibr7-17449871261419412] Fond G Lucas G Boyer L (2023b) Untreated major depression in healthcare workers: Results from the nationwide AMADEUS survey. Journal of Clinical Nursing 32: 7765–7772. DOI: 10.1111/jocn.16673. 36949278

[bibr8-17449871261419412] Gaddour A Nakhli R Chouchane A , et al. (2025) Long‑term psychological impact of the COVID‑19 pandemic on healthcare workers. European Psychiatry 68: S623–S624. DOI: 10.1192/j.eurpsy.2025.1270.

[bibr9-17449871261419412] Gomes S Santos MM Carolino ET (2013) Psycho-social risks at work: Stress and coping strategies in oncology nurses. Revista Latino-Americana de Enfermagem 21: 1282–1289. DOI: 10.1590/0104-1169.2742.2365. 24271316

[bibr10-17449871261419412] Gray J Grove S (2021) The Practice of Nursing Research, 9th ed. Amsterdam: Elsevier.

[bibr11-17449871261419412] Pinho LG Sampaio F Sequeira C , et al. (2021) Portuguese nurses’ stress, anxiety, and depression reduction strategies during the COVID19 outbreak. International Journal of Environmental Research and Public Health 18: 3490. DOI: 10.3390/ijerph18073490. 33801740 PMC8037799

[bibr12-17449871261419412] Lim H Kim SH (2025) Navigating night shifts: A qualitative study of exploring sleep experiences and coping strategies among nurses. BMC Nursing 24: 1–11. DOI: 10.1186/s12912-025-03001-3. 40197250 PMC11978025

[bibr13-17449871261419412] Lucas P Jesus É Almeida S , et al. (2025) The nursing practice environment and job satisfaction, intention to leave, and burnout among primary healthcare nurses: A cross-sectional study. Nursing Reports 15: 224. DOI: 10.3390/nursrep15070224. 40710919 PMC12300685

[bibr14-17449871261419412] Ma T-T Wang Z Qin X , et al. (2023) Global trends in the consumption of benzodiazepines and Z-drugs in 67 countries and regions from 2008 to 2018: A sales data analysis. Sleep 46: zsad124. DOI: 10.1093/sleep/zsad124. 37094086

[bibr15-17449871261419412] Meneses RF Barros C Sousa H , et al. (2024) Risks of ecosystems’ degradation: Portuguese healthcare professionals’ mental health hope and resilient. Sustainability 16: 5123. DOI: 10.3390/su16125123.

[bibr16-17449871261419412] Min A Hong HC (2022) Work schedule characteristics associated with sleep disturbance among healthcare professionals in Europe and South Korea: A report from two cross-sectional surveys. BMC Nursing 21: 1–9. DOI: 10.1186/s12912-022-00974-3. 35850698 PMC9290258

[bibr17-17449871261419412] Missouridou E (2017) Secondary posttraumatic stress and nurses’ emotional responses to patient’s trauma. Journal of Trauma Nursing 24: 110–115. DOI: 10.1097/JTN.0000000000000274. 28272184

[bibr18-17449871261419412] Moya-Salazar J Jaime-Quispe A Cañari B , et al. (2023) A systematic review of mental health in rural Andean populations in Latin America during the COVID-19 pandemic. Frontiers in Psychiatry 14: 1136328. DOI: 10.3389/fpsyt.2023.1136328. PMC10470633 37663592

[bibr19-17449871261419412] OECD/European Observatory on Health Systems and Policies (2023) Portugal: perfil de saúde do país 2023. State of Health in the EU. OECD Publishing. Available at: https://www.oecd.org/content/dam/oecd/pt/publications/reports/2023/12/portugal-country-health-profile-2023_eebec3f5/6be7d83c-pt.pdf

[bibr20-17449871261419412] OECD (2025) Health at a glance 2025: OECD indicators. OECD Publishing. DOI: 10.1787/8f9e3f98-en.

[bibr21-17449871261419412] Paguio JT Yu DS Su JJ (2020) Systematic review of interventions to improve nurses’ work environments. Journal of Advanced Nursing 76: 2471–2493. DOI: 10.1111/jan.14462. 32770584

[bibr22-17449871261419412] Pais-Ribeiro JL Neto C Silva M , et al. (2015) Ulterior validação do questionário de saúde geral de Goldberg de 28 itens. Psicologia. Saúde e Doenças 16: 278–285. Available at: https://www.scienceopen.com/document?vid=58f740b5-364c-42a8-a141-c24415b244c3

[bibr23-17449871261419412] Pereira H Fehér G Tibold A , et al. (2021) The impact of shift work on occupational health indicators among professionally active adults: a comparative study. International Journal of Environment Research and Public Health 18: 11290. DOI: 10.3390/ijerph182111290. PMC8583436 34769807

[bibr24-17449871261419412] Ramos-Toescher AM Tomaschewisk-Barlem JG Barlem EL , et al. (2020) Saúde mental de profissionais de enfermagem durante a pandemia de COVID-19: Recursos de apoio. Escola Anna Nery 24: e20200276. DOI: 10.1590/2177-9465-EAN-2020-0276.

[bibr25-17449871261419412] Ring M Hult M (2025) A structural equation model of the impacts of nurses’ psychological safety and psychological contract breach. Journal of Advanced Nursing 81: 1323–1331. DOI: 10.1111/jan.16331. 39003643

[bibr26-17449871261419412] Sagherian K Steege L Cho H , et al. (2020) Global prevalence of burnout symptoms among nurses: A systematic review and metaanalysis. Journal of Advanced Nursing 76: 1121–1134. DOI: 10.1111/jan.14423.

[bibr27-17449871261419412] Sampaio F Sequeira C Teixeira L (2020) Nurses’ mental health during the COVID19 outbreak: A cross-sectional study. Journal of Occupational and Environmental Medicine 62: 783–787. DOI: 10.1097/JOM.0000000000001987. 32769803

[bibr28-17449871261419412] Seabra PR Lopes JO Calado M , et al. (2019) A national survey of the nurses’ mental health: The case of Portugal. Nursing Forum 54: 425–433. DOI: 10.1111/nuf.12350. 31055850

[bibr29-17449871261419412] Seabra PR Capelas ML Lopes JO , et al. (2021) Psychometric properties of the 28-item general health questionnaire in nurses: A proposal with 24 items. Revista de Enfermagem Referência 5: e20136. DOI: 10.12707/RV20136.

[bibr30-17449871261419412] Squires A Applebaum B Rojas-Guyler L , et al. (2025) Global priorities in nurse well-being: Lessons from the COVID-19 pandemic. International Nursing Review 72: 5–14. DOI: 10.1111/inr.12857.

[bibr31-17449871261419412] Silva S Borges E Abreu M , et al. (2016) Relação entre resiliência e burnout: Promoção da saúde mental e ocupacional dos enfermeiros. Revista Portuguesa de Enfermagem de Saúde Mental 16: 41–48. Available at: https://repositorio.usp.br/item/002869182

[bibr32-17449871261419412] Moura RC Chavaglia SR Coimbra MA , et al. (2022) Transtornos mentais comuns em profissionais de enfermagem de serviços de emergência. Acta Paulista de Enfermagem 35: eAPE03032. Available at: https://www.scielo.br/j/ape/a/wHvYRr4Q7M7p5bKyDmCpZjP

[bibr33-17449871261419412] Vala KC Fishbein JS Bellehsen MH , et al. (2025) Patterns of mental health and resilience among nurses and physicians throughout the COVID-19 pandemic: A 3-year longitudinal study. Journal of Occupational and Environmental Medicine 67: e152–e157. DOI: 10.1097/JOM.0000000000003291. PMC11895821 39639521

[bibr34-17449871261419412] Vandenbroucke JP Von Elm E Altman DG , et al. (2007) Strengthening the reporting of observational studies in epidemiology (STROBE): Explanation and elaboration. PLoS Medicine 4: 1628–1654. DOI: 10.1371/journal.pmed.0040297. PMC2020496 17941715

[bibr35-17449871261419412] Yan Y Zhao C Bi X , et al. (2025) The mental workload of ICU nurses performing human-machine tasks and associated factors: A cross-sectional questionnaire survey. Journal of Advanced Nursing 81: 224–236. DOI: 10.1111/jan.16199. 38687803

[bibr36-17449871261419412] Yuan Z Wang J Feng F , et al. (2023) The levels and related factors of mental workload among nurses: A systematic review and meta-analysis. International Journal of Nursing Practice 29: e13148. DOI: 10.1111/ijn.13148. 36950781

[bibr37-17449871261419412] Zhang A Gagné T Walsh D , et al. (2023) Trends in psychological distress in Great Britain. 1991-2019: Evidence from three representative surveys. Journal of Epidemiology and Community Health 77: 468–473. DOI: 10.1136/jech-2022-219660. 37188500 PMC10313989

[bibr38-17449871261419412] Zhang L Wen J Yuan L , et al. (2025) Anxiety and depression in healthcare workers 2 years after COVID-19 infection and scale validation. Scientific Reports 15: 13893. DOI: 10.1038/s41598-025-98515-w. 40263530 PMC12015455

